# Supplementary material for: A Comparative Study of the Resilience of Urban and Rural Areas under Climate Change
Source: Int J Environ Res Public Health. 2022 Jul 22;19(15):8911. doi: 10.3390/ijerph19158911 (PMC9331052; doi:10.3390/ijerph19158911)
Supplement: Supplementary file 1 [file ijerph-19-08911-s001.zip › ijerph-1791159-Supplementary.pdf]

Supplemental File

Supplemental Table S1. The calculation method and data source of the indicator.

Orientation	Indicator	Calculation	Source	Data type
Social	Aging index	Population over 65 years old/population 0-14 years old	Social and economic information service platform of the census and statistics department (2019)	Statistical data
	Education level	Population with high school (inclusive) and above/total population		Statistical data
	Household size	Average household population		Statistical data
	Dependency ratio	Population aged 0-14 and over 65/population aged 15-64		Statistical data
	Population density	Population/area		Statistical data
	Disabled population	Disabled population/total population		Statistical data
Economic	Agricultural land area	Agricultural land area/total area	Land use survey of the center for land surveying and mapping (2016)	Graphic data
	Number of agricultural households	Number of agricultural households/total number of industrial and commercial households	Social and economic information service platform of the census and statistics department (2019)	Statistical data
	Residence income	Total local income/total population	Comprehensive income tax return (2018)	Statistical data
	Low-income households	Number of low-income households/total population	Social and economic information service platform of the census and statistics department (2019)	Statistical data
Infrastructure	Medical facilities	Emergency medical and first-aid hospital covered area/total area after buffer 5 km	Emergency medical responsibility hospital in chiayi region (2019)	Point information
	School	Covered area/total area after 800 m of cultural and educational facilities buffer	Directory of schools at all levels of the census and statistics department (2019)	Point information
	Fire station	Covered area/total area of fire station buffer after 2700 m	Chiayi county fire station (2019)	Point information
	Road density	Road land area/total area	Land use survey of the center for land surveying and mapping (2016)	Graphic data
Environment	Impervious area	Building land/total area	Disaster potential map of disaster prevention and rescue technology center (2019)	Graphic data
	Green infrastructure	Park and wetland area/total area		Graphic data
	Green area	Farmland and forest area/total area		Graphic data
Disaster threat	Earth-rock flow potential	Potential area of earth-rock flow/total area	Land use survey of the center for land surveying and mapping (2016)	Graphic data

	Stratum subsidence	Severe stratum subsidence area/total area		Graphic data
	Landslides	Landslide and ground slip area/total area		Graphic data
Traditional knowledge	Percentage of indigenous population	Indigenous population/total population	Social and economic information service platform of the statistics department (2019)	Statistical data
	Proportion of aboriginal elderly population	Indigenous population over 65 years old/indigenous population		Statistical data
